# Elemental profiles in distant tissues during tumor progression

**DOI:** 10.1186/s12885-023-10782-w

**Published:** 2023-04-06

**Authors:** Samella Salles, Rebecca Salles, Mauro S. G. Pavão, Simone C. Cardoso, Mariana P. Stelling

**Affiliations:** 1 Federal Institute of Education, Science and Technology of Rio de Janeiro (IFRJ), Rio de Janeiro, Brazil; 2 Federal Center of Technological Education (CEFET/RJ), Rio de Janeiro, Brazil; 3 Medical Biochemistry Institute, Federal University of Rio de Janeiro (UFRJ), Rio de Janeiro, Brazil; 4 Physics Institute, Federal University of Rio de Janeiro (UFRJ), Rio de Janeiro, Brazil

**Keywords:** Tumor progression, Essential mineral elements, Lewis lung carcinoma, Elemental distribution, SR-XRF

## Abstract

**Background:**

Essential elements have functions in tumor progression by promoting protumoral cellular processes, such as proliferation, and migration, among others. Obtaining an understanding of how these elements relate to tumor progression processes is of great importance for research. Elemental profile studies in distant tissues, which can be modulated by tumor cells to promote metastasis, have not been sufficiently investigated. The main goal of this study is to evaluate multielemental distribution during tumor progression, focusing on tumor tissue and distant tissues that may be affected.

**Methods:**

Tumor progression in vivo was simulated by inoculating C57BL/6 mice with Lewis Lung Carcinoma (LLC) cells. Samples of the primary tumor and distant tissues were collected during 5 weeks of tumor progression for the control and experimental (tumor-bearing) groups. The biological samples were analyzed using the synchrotron radiation X-Ray fluorescence technique. Data on the concentration of P, S, K, Ca, Mn, Fe, Cu, and Zn in the samples were obtained and statistically analyzed to evaluate the distribution of the elements during tumor progression in the primary tumor as well as distant tissues.

**Results:**

It was possible to observe significant changes in the concentrations’ distribution of P, S, K, Ca, Mn, Fe, and Cu in distant tissues caused by the presence of tumor cells. It was also possible to detect a greater similarity between tumor tissue (which has the lung as tissue of origin) and a tissue of non-origin, such as the liver, which is an unprecedented result. Moreover, changes in the distributions of concentrations were detected and studied over time for the different tissues analyzed, such as primary tumor, liver and lung, in Control and Tumor groups.

**Conclusions:**

Among other results, this paper could explore the modulation of distant tissues caused by the presence of a primary tumor. This could be achieved by the evaluation of several elements of known biological importance allowing the study of different biological processes involved in cancer. The role of essential elements as modulators of the tumor microenvironment is a relevant aspect of tumor progression and this work is a contribution to the field of tumoral metallomics.

**Supplementary Information:**

The online version contains supplementary material available at 10.1186/s12885-023-10782-w.

## Background

Essential mineral elements are crucial for life, being necessary for numerous biological processes in the human body. It is well known that such elements can also have functions in tumor progression, promoting its cellular division, migration, survival, angiogenesis, maintaining cellular integrity, among other processes [1–4].

Knowing how cancer develops, progresses, and spreads (metastasis), including how cancer cells interact with normal cells and tissues in other parts of the body, is required to mobilize the development of new ways to prevent, detect, diagnose and treat cancer [5]. Understanding how the role of mineral elements is related to the processes of tumor progression is of great relevance for research.

The literature presents research regarding the relationship between chemical elements and cancer. They focus mainly on the determination of elements’ concentrations in different types of tumor to characterize them [6, 7]; the comparison between distinct tissues and cells, such as normal and tumor tissues [8], tumor cells and surrounding healthy cells [9], normal, benign and malignant tissues [10], primary tumor and metastases [11]; the study of elements collected from the environment that could be related to the incidence of cancer [12–14]; the investigation of antitumoral molecules [15, 16]; as well as the evaluation of techniques of elemental analysis [17–19].

Research, such as the works of Naidu et al. [8], Farquharson et al. [9], Liu et al. [10] and Al-Ebraheem et al. [11], showed significant changes in the elemental profiles between the different tissues analyzed, which emphasize the relevance of exploring the tumoral metallomics.

However, comparative studies of the elemental profile between tumors and distant tissues are still scarce. It is known that the tumor can actively prepare distant organs before the arrival of cancer cells during the metastasis process, which could facilitate invasion and colonization by tumor cells [20, 21]. Hence, the role of elements in tumor progression and the elemental profile in processes such as distant tissue modulation is a subject of great relevance that presents research opportunities.

Among the comparative studies regarding distant tissues, the work of Frank et al. from 1986 [22], establishes the elements’ profile during tumor progression of Lewis Lung Carcinoma (LLC) cells. They observed the elements and their fluctuations during tumor growth in the tumor tissue as well as lung, liver, and skeletal muscles, through the X-Ray fluorescence technique induced by radioisotopes. The paper highlighted the presence of large fluctuations in the elements’ levels throughout tumor progression, in addition to the elements profile of the primary tumor greatly resembling its original tissue (the normal/healthy lung) and being very different from other normal tissues studied.

In this context, there is currently a demand for progressing and updating the study on elemental profile in tumors and distant tissues that can be modulated by tumor cells to promote metastasis. This work addresses this demand by evaluating the elemental distribution of concentrations during tumor progression, focusing not only on the tumor tissue but also on distant tissues. The role of several elements of known biological importance (P, S, K, Ca, Mn, Fe, Cu, and Zn) are evaluated, allowing the study of different processes of cancer [2–4].

For this work, we analyzed data indicating the presence, concentration, and location of different elements in tissues such as the primary tumor and distant tissues, in control and experimental (tumor-bearing) groups, obtained in 5 weeks of tumor progression. The simulation of tumor progression in vivo was obtained via the inoculation of C57BL/6 mice (isogenic) with LLC cells (murine Lewis Lung Carcinoma). This tumor cell line has the lung as its tissue of origin and is widely used as a model of metastasis, being tumorigenic. They are known to present tropism to the lungs, especially and, in some cases, the liver, which makes this cell line able to cause metastasis to such organs [23–28]. The analysis of the biological samples was performed through the synchrotron radiation X-Ray Fluorescence technique. Such a technique has been recognized as an ideal tool to reliably and simultaneously determine the elemental distribution in tissues, detecting the presence, quantity, and location of elements, with reasonable sensitivity, specificity, and resolution [29, 30]. Hence, there are many studies where X-Ray Fluorescence is used to investigate the role elements play in different diseases, such as cancer, and tissues, such as normal and tumor tissues [8–10, 31–33]. The data harvested in this research can reveal the multielemental composition of the primary tumor and distant tissues, such as the lung and liver. In order to extract relevant information inherent to the voluminous data available, data preprocessing and statistical analyses were performed.

With this work, it was possible to observe significant changes in the elemental concentrations’ distribution in distant tissues caused by tumor cells. It was also possible to detect the similarity between tumor tissue and a tissue of non-origin, which is an unprecedented result. Moreover, time-dependent elemental changes were analyzed and compared in this work for the different tissues and groups studied. Finally, the importance of elements for biological processes of normal cells, as well as tumor cells during their tumor progression was also discussed.

## Methodology

This research was conducted in two phases: biological experiment ([34]) and data analysis.

### Biological Experiment

#### Cell Culture

The LLC cells (murine Lewis Lung Carcinoma, ATCC) were cultured under standard conditions at 37$^{\circ }$C and CO$_2$ 5% atmosphere. DMEM (Vitrocell) cell culture medium was supplemented with fetal bovine serum (FBS) 10% (v/v) (Sigma-Aldrich) and 4.5 mg/L glucose (Sigma-Aldrich). Cells were passaged regularly with a trypsin-EDTA solution (0.25% trypsin and 1mM EDTA).

#### Animal Model of Spontaneous Metastasis

C57BL/6 male and female mice, aged between 8 and 12 weeks, were used as animal models. To simulate tumor progression in vivo, these animals were anesthetized and inoculated subcutaneously in the posterior dorsolateral region with $5\times 10^5$ LLC cells in 60 $\upmu$L serum-free DMEM as vehicle. The animals just described are part of the tumor-bearing group, from now on called the tumor group. The control animals (control group) were inoculated only with the vehicle.

All animals were kept under standard conditions with access to food and water ad libitum and were monitored for their vital signs, behavior, and tumor size from 0 to 5 weeks after LLC cell inoculation.

Tissue samples (liver, lung, and primary tumor) were collected. The primary tumor (originated from LLC cells inoculated in the tumor group animals) and the liver had their samples collected at weeks 0, 1, 3, and 5 allowing for the study of tumor progression in a time-dependent manner. The lung had its samples collected only at week 5, since this tissue is known as the main organ to which LLC cells migrate (already discussed in this section) and could cause alterations in elemental distribution, which could be detected during the more advanced weeks of tumor progression for the process of metastasis [20, 21, 23–28].

The collected samples were washed with PBS, briefly and superficially fixed in 4% paraformaldehyde for 5 min, and cryopreserved in OCT (Optimal Cutting Temperature compound - Tissue-Tek) for further sectioning in cryostat (Leica-CM1850- Germany).

All procedures involving animal experimentation were approved by the Federal University of Rio de Janeiro Animal Experimentation Committee (protocol number: 015/18) and were performed in accordance with the Brazilian guidelines for scientific use of animals.

#### Synchrotron Radiation Induced X-Ray Fluorescence Technique

The X-ray fluorescence technique (described in section Background) was performed at the UVX Light Source (at D09B X-ray Fluorescence beamline) at the Brazilian Synchrotron Light Source Laboratory (LNLS).

Tissue samples were cryosectioned into 20 $\mu$m-thick slices and placed on Ultralene film. Samples were excited by a white beam with energy ranging from 5 keV to 22 keV. A silicon drift detector (KETEK GmbH) with 140 eV (FWHM) at 5.9 keV placed at 90$^{\circ }$ from the incident beam was used to collect X-ray fluorescent and scattered radiation coming from samples. An optical system based on a pair of bent mirrors in a Kirkpatrick-Baez arrangement was used to focus the X-Ray beam down to 20 $\mu$m spatial resolution. Each spot was irradiated for 1s in fly scan mode. The total irradiated area varied from 100 to 400 $\mu$m2, depending on the sample. Elemental content was assessed by total photon count normalized by the number of irradiated spots. The data were fitted using fundamental parameters method [34, 35].

For more details of the biological experiment refer to the work of Stelling et al. [34].

### Data Analysis

After the execution of the biological experiment, the data obtained were prepared for analysis, going through the preprocessing step. This phase involves cleaning, transformation, integration, and data selection [36].

#### Data Preprocessing

The data obtained from the biological experiment contain, for each tissue, group and week analyzed, values in mass fraction for each element detected at each point/location of each sample.

Data cleaning was carried out, where data referring to elements affected by artifacts, such as argon, an element within detection range and present in the atmosphere, were filtered and removed from the data for analysis. The mass fraction values were transformed to ppm concentration. Next, data files were integrated generating a single dataset containing 13 attributes regarding Group, Tissue, Week, Sample (animal), and Point, in addition to each of the eight elements (P, S, K, Ca, Mn, Fe, Cu, and Zn). The integrated dataset features over 188,000 points detected for each element.

Table 1 shows the amount of samples and point data acquired for each Group, Tissue and Week analyzed. For instance, for week 5 of the Primary Tumor (Tumor group), 14 samples were studied, which resulted in 30504 points to be analyzed for each element.

**Table 1:** Summary of the amount of data acquired for each Group, Tissue and Week

Group	Tissue	Week	Samples	Points
Control	Liver	0	3	15444
Control	Liver	1	3	11675
Control	Liver	3	5	16415
Control	Liver	5	7	22885
Control	Lung	5	7	5188
Tumor	Liver	1	4	9097
Tumor	Liver	3	6	19467
Tumor	Liver	5	8	33343
Tumor	Lung	5	6	7456
Tumor	Primary Tumor	1	5	4092
Tumor	Primary Tumor	3	8	13410
Tumor	Primary Tumor	5	14	30504
Total			76	188976

Finally in the preprocessing step, the data could be selected according to the research objectives. Therefore, the attributes “Sample” (animal), and “Point” were removed. The attribute “Point” was discarded since it was not possible to compare the locations of the elements in different samples. Such attribute (points) can be used in the construction of spatial distribution maps (imaging technique) however, this is not the focus of the current work. The sample attribute could be removed, considering that the animals in question are isogenic and were kept under the same conditions during the biological experiment performed.

#### Statistical Analysis

**Fig. 1:**
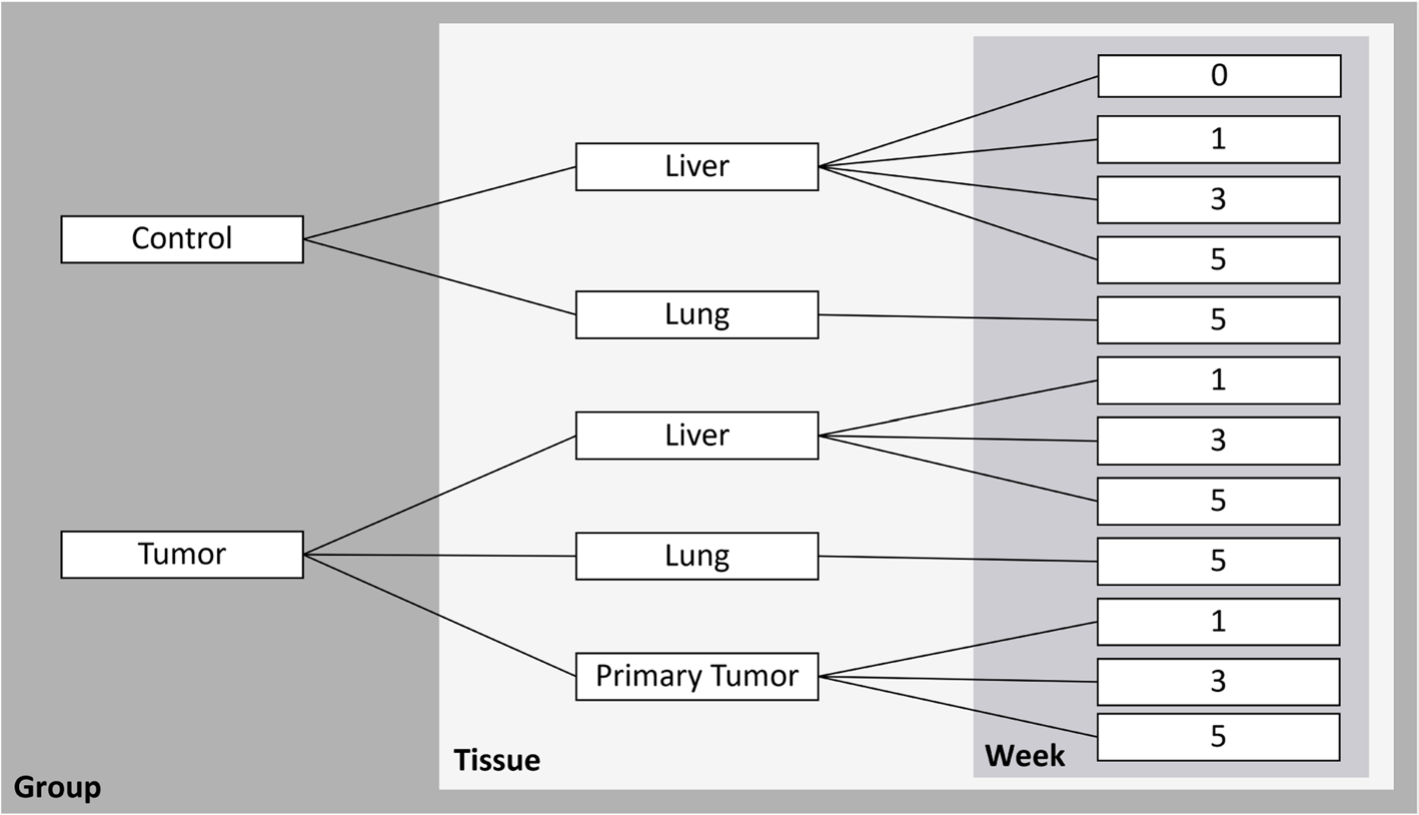
Groupings created for analysis. The different shades of grey highlight the three different groupings created, such as Group, Tissue, and Week level, from most broad to more detailed level

During the statistical analysis, groupings were created to allow comparisons within the data to be made from a broader to a more detailed level. Thus, groupings were obtained at the group level, at tissue level, and week level, as seen in Fig. 1. The first one contains data from all tissues and weeks for each group, that is, control and tumor. The second one presents data from all weeks for each tissue within each group. Finally, the week level refers to data for each week in each tissue and group.

This work uses the week level grouping (more detailed grouping) for analysis to allow the observation of any changes in the distribution of elements that occur over time. Also, in order to make comparisons between all groups and tissues, week 5 (seen in the week level) was used since this week possesses data for all tissues and groups (Fig. 1). Thus at the week level, one can compare tissues at week 5 of each specific group, such as the liver and lung tissues of the control group, or even compare tissues from different groups, such as the liver of the control group versus liver of the tumor group. Hence, with this grouping is possible to compare weeks of different tissues and groups or even follow the weeks within the same tissue, observing changes over time. To make time-dependent comparisons, weeks that have data in both groups were used, hence weeks 1, 3, and 5.

Our research data present a non-normal distribution since the concentrations distribution of each element along the tissue is asymmetric. That is, very different concentration values can be detected because, in the same tissue sample, there can be high and very low elemental concentrations in different locations. Thus, the selection of statistical measures and relevance tests resistant to such asymmetry is necessary [37].

In order to obtain statistical summaries of the concentration data, the median and interquartile range were used. The Wilcoxon test (with 95% confidence) was chosen to analyze the statistical relevance of the differences between the concentration distributions. This test stands out from others as it is resistant to non-normal distribution and can still be used for log data [38].

Both preprocessing step and data analysis were carried out via the R language and computational environment. R is one of the main languages for statistical data analysis, including its preprocessing [39]. The data obtained from the experiment and the code used in the preprocessing and analysis of these are available at GitHub^1^.

## Results

**Fig. 2:**
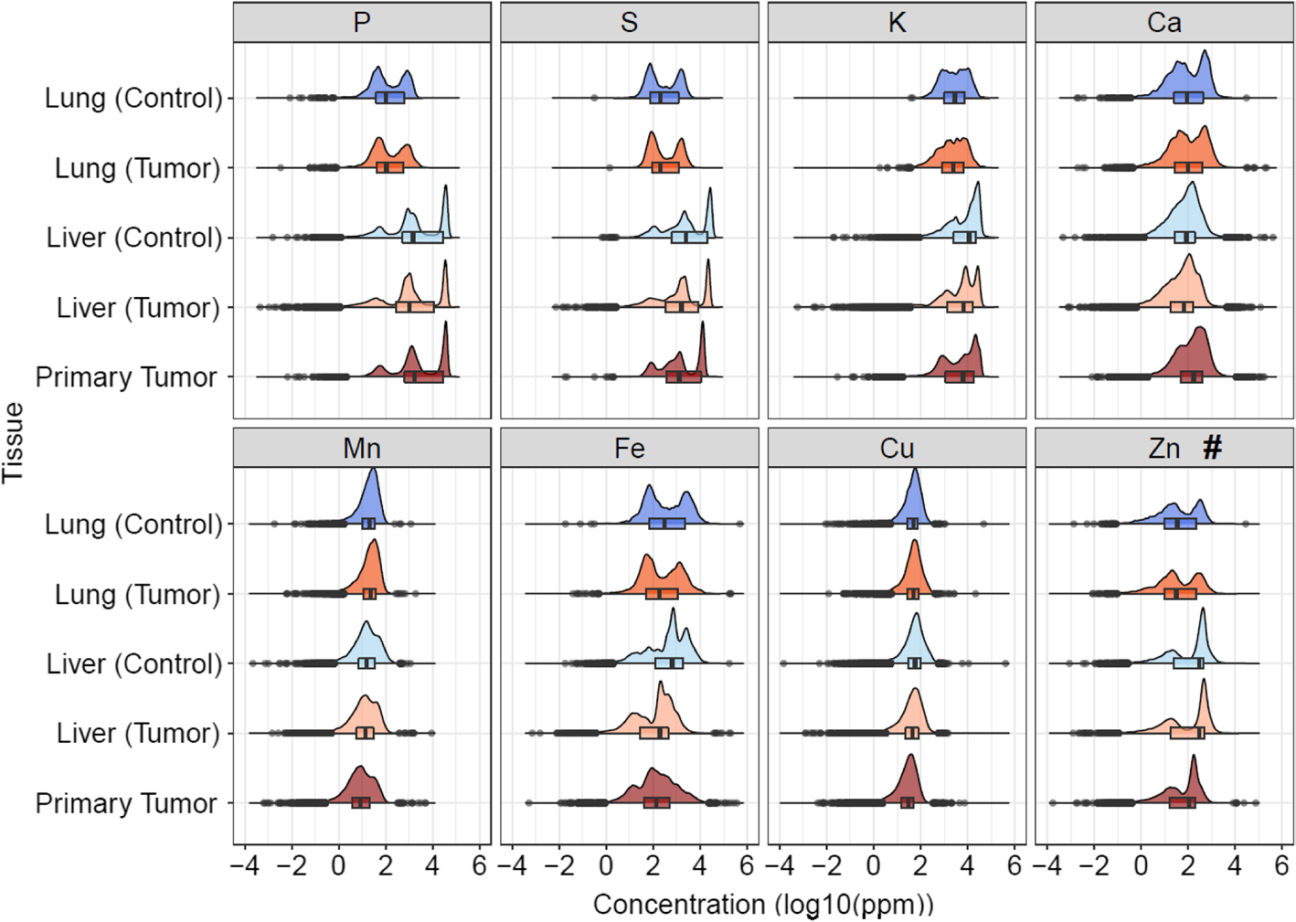
Comparison of elements distributions, at week 5, in all tissues and groups analyzed. The density plots in shades of blue represent the Control group and those in shades of red the Tumor group. Tissues can be seen on the left of the plot, where liver, lung, and primary tumor are seen. The symbol # represents the only element showing not statistically significant differences between their groups’ distributions

Figure 2 shows elements’ concentrations distribution in the tissues (primary tumor, liver and lung) for the Control and Tumor groups at 5^th^ week of tumor progression. This figure shows boxplots and density plots corresponding to the distribution. In these plots, the greater the frequency of concentration values in that range, the greater the peak of the density plot observed.

With this grouping (at the week level), it’s possible to observe changes that occurred in the tissues between the two groups and it becomes possible to detect alterations in distant tissues caused by the presence of the primary tumor.

It is possible to highlight that 7 of the 8 elements obtained a statistically significant difference in their distributions between the groups (see table 2). Zinc (Zn) (underlined in Table 2) was the only one that presented not statistically significant differences in its distributions between Control and Tumor.

**Table 2:** *P*-Values indicating the significance of the statistical difference between the elements distribution of Control and Tumor groups for the Lung and Liver tissues, at week 5

Element	Tissue	
	Lung	Liver
P	$2,24\times 10^{-2}$	$4,17\times 10^{-157}$
S	$2,01\times 10^{-9}$	$9,40\times 10^{-294}$
K	$1,07\times 10^{-14}$	$5,46\times 10^{-284}$
Ca	$3,97\times 10^{-2}$	$2,86\times 10^{-96}$
Mn	$1,68\times 10^{-4}$	$4,93\times 10^{-70}$
Fe	$1,68\times 10^{-43}$	0
Cu	$9,21\times 10^{-4}$	$1,96\times 10^{-177}$
Zn	$\underline{6,11\times 10^{-1}}$	$\underline{1,65\times 10^{-1}}$

When analyzing Fig. 2 it is noticeable that, for most elements, the distributions present considerable differences between liver and lung tissues. In addition, a greater similarity between the primary tumor and the liver can be observed when compared to the primary tumor and lung. Furthermore, when comparing the liver and lung distributions between their Control and Tumor groups at week 5, it can be observed that while the lung does not seem to be much altered, the liver appears to show more differences between Control and Tumor. Iron (Fe) also stands out since it presents greater differences in its distributions between tissues and groups. Copper (Cu), although still presents relevant differences between its tissues and groups, shows some stability in its distributions, when compared to the other elements.

**Fig. 3:**
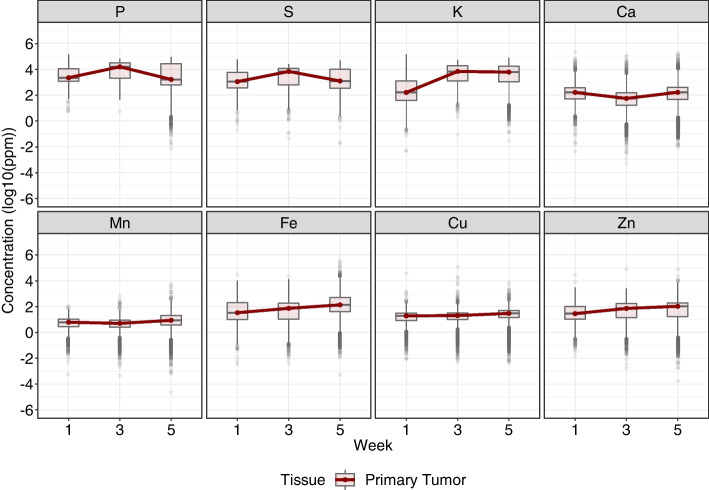
Comparison of the elements’ distributions throughout the weeks (1, 3, and 5) in the primary tumor. Boxplots represent the distribution of each element throughout the weeks. The medians of concentration for each week are emphasized

In addition to observing the primary tumor against the other analyzed tissues, it was also possible to focus only on the tumor tissue. Figure 3 shows boxplots that represent the distribution of each element throughout the weeks, in which the medians of concentration for each week are emphasized. Hereby, it was possible to observe elements with distinct distributions, presenting alterations in their distribution for each week. It can be highlighted the increase in the level of iron (Fe) over time, the increase in the level of potassium (K) from week 1 to 3, in addition to the elements sulfur (S) and phosphorus (P) showing similarities between their levels of concentration and behavior throughout the weeks.

**Fig. 4:**
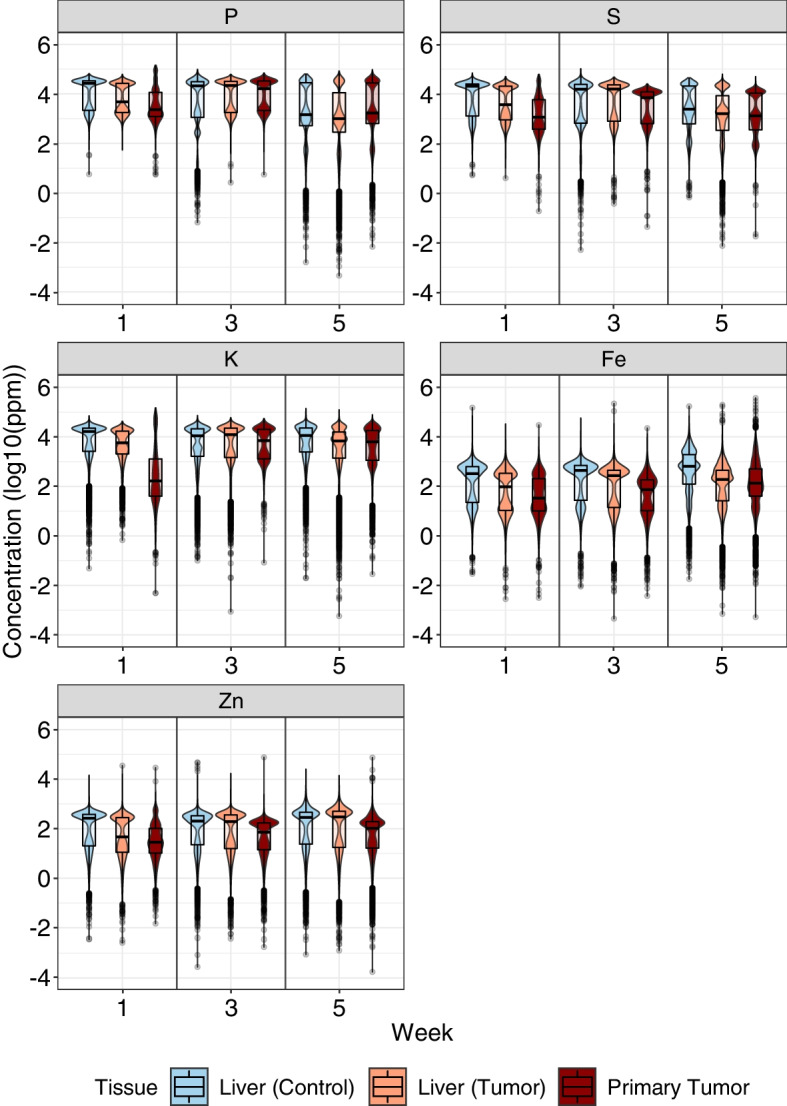
Comparison of the elements’ distributions throughout the weeks (1, 3, and 5) in the liver (Control and Tumor groups) and primary tumor. Boxplots and violin plots in which light colors represent the liver, with light blue showing the Control group and light red the Tumor group. The primary tumor is represented in dark red

Figure 4 shows boxplots and violin plots that illustrate the concentration distributions of the elements in the liver, throughout the weeks (1, 3, and 5), for the Control and Tumor Groups. These data were also compared with those obtained for the tumor tissue. Thus, with this figure, we can observe alterations, caused by the presence of the primary tumor, in the liver (distant tissue) over time, in addition to observing possible similarities between these two tissues. This figure showed the most relevant elements for discussion. To observe all 8 elements in more detail, see Additional file 1 (section 5).

In Fig. 4, the elements phosphorus (P) and sulfur (S) seem to have both their concentration values and distributions similar. Over the weeks, the distributions of these two elements also show similarities. With the statistical analysis performed, it was also possible to notice that zinc (Zn) presents its distributions with no statistically relevant differences in the liver, between Control and Tumor Groups, in weeks 3 and 5 (*p*-values of $4.32\times 10^{-1}$ and $1.65\times 10^{-1}$, respectively).

In addition to investigating how the elements had their distribution modified over time between the two groups, we also looked for similarities between the liver (Tumor Group) and the primary tumor. Interestingly, in Fig. 4, it was observed that potassium (K), while presenting distributions with statistically significant differences between the Control and Tumor groups, as seen in Fig. 2, had its distributions showing smaller differences between liver (Tumor Group) and the primary tumor over the weeks, where at week 5 there were not statistically relevant differences in the distributions between these two tissues (*p*-values of 0, $6.05\times 10^{-25}$ and $7.46\times 10^{-2}$ from week 1 to 5).

## Discussion

The first question we sought to answer is regarding the existence of alterations in elemental distributions in distant tissues. As approached in section Background, distant tissues are described in the literature as primary tumor targets for the formation of pre-metastatic niches that could facilitate invasion and colonization by tumor cells during metastasis [20, 21]. When evaluating the differences in the distribution of elements between the groups (Control and Tumor) for the liver and lung (Fig. 2), it was possible to notice that, except for zinc, all elements obtained statistically significant differences in their distributions. Since the most relevant difference among the analyzed groups is the subcutaneous inoculation of LLC cells that developed into a tumor (Tumor group), with this work, it is possible to observe statistically significant changes in the distribution of elements in distant tissues caused by the presence of the tumor, which could be indicative of distant tissue modulation.

Another aspect explored in this work was concerning whether the tumor showed any particular preference for modulation for one of the distant tissues. According to the literature, among the liver and lung, LLC cells have a preference for the lung, when inoculated *ex-situ*, during the metastasis process, which can be attributed to its lung epithelial origin [26–28]. However, considering both distant tissues presented such statistically significant differences in their distributions between the Control and Tumor groups, this work can not highlight a preference for tumoral modulation for a tissue in particular.

When analyzing elements’ concentrations distribution in all tissues (Fig. 2) at week 5, it was possible to observe a greater similarity between the liver and the primary tumor, compared to the tumoral tissue and lung. This fact is unexpected due to the LLC lineage pulmonary origin. Moreover, the similarity of this lineage type with the tissue of origin but differences with other tissues have already been established in the work by Frank et al. [22]. While researching for bibliographies that address such similarity between tumoral tissue and liver, it was not possible to find such a result in other works. Thus, such a result showed to be unprecedented.

Focusing on the differences between the distributions of the elements (as seen in Fig. 2), in addition to zinc (Zn), copper (Cu) also showed fewer variations in their distributions among different groups and tissues. Although copper (Cu) still showed a statistically significant difference between Control and Tumor. This makes sense since these two elements (zinc and copper) are of great importance to cells and are closely related. These two elements have known essential functions such as: maintaining the integrity of the cell membrane; cell proliferation and growth; having antioxidant properties, by participating in the structure of the antioxidant enzyme superoxide dismutase (SOD), guaranteeing defense against reactive oxygen species (ROS) and consequently also maintaining the integrity of the DNA; being involved in gene expression, apoptosis, among other processes [40–49]. Due to such functions and their importance, the level of these elements and their transport are tightly regulated [2].

In contrast with the two elements above, iron was an element that showed noticeable variations in its distribution over the weeks for groups and tissues. Despite each tissue having its specific need for this element, in the Figs. 2 and 4 a greater variation in iron distributions between the Control and Tumor groups of the liver can be seen, in addition to an apparent increase of this element, over the weeks in the tumor tissue, also observed in the Fig. 3.

Iron is essential in a vast number of biological phenomena [2]. This element controls cell proliferation and growth by regulating DNA synthesis, besides having a role in the tumor microenvironment, angiogenesis, and metastasis [1, 50–52]. Therefore, this element is important for tumor progression, the formation of pre-metastatic niches, and the colonization of tissues. Since tumor cells present excessive proliferation rates, it is believed that tumor cells require higher concentrations of this element. Consequently, higher concentrations of iron are often observed in tumor tissues, in which there are increases and decreases in the expression of proteins involved in iron uptake and its efflux [50, 51, 53, 54]. This increase can be seen in Figs. 3 and 4, where the median of iron concentrations (seen in the boxplot) increase over the weeks.

It is important to highlight that every tissue has its specific needs and functions, which may require different concentrations and distributions of elements, even throughout the weeks, especially during tumor progression [55]. Thus, when we focus on the primary tumor (Fig. 3), greater differences can be observed between the distributions over the weeks. This result is consistent with the literature and with what has been described, since the tumor tissue is developing, adjusting to its environment, and reciprocally modulating it [56]. Thereby, the changes in the distribution of the elements in this tissue are part of the stages of tumor progression, such as the increase in iron (Fe) levels throughout time within this tissue [50]; the increase in zinc (Zn) levels, which is already known to be captured from the circulation by tumor cells to maintain their growth and the integrity of their membrane [46, 48]; in addition to the increase in potassium level in the initial weeks of tumor progression, as will be explored next [57].

In Fig. 4, when we focus on the liver and compare it with the primary tumor, looking for signs of alterations in this distant tissue over time, an element stands out. The statistical analysis performed showed that potassium (K) had its distributions with statistically relevant differences between the Control and Tumor groups in all weeks analyzed. However, this element had its distributions between liver (Tumor Group) and primary tumor showing smaller differences over the weeks (Fig. 4), obtaining no statistically relevant differences at week 5. This fact is interesting since the tumoral tissue could be modulating the levels of potassium in the distant tissue (liver) during tumor progression to levels different from Control, but similar to the primary tumor in itself. The importance of this element for cell functions and tumoral progression, especially for metastasis could explain its modulation in the tissue. When discussing potassium’s role, it is essential to acknowledge potassium ion channels (the most abundant type of ion channel)[58, 59]. These channels are crucial for the proper functioning of cells, including tumor cells, besides being important for the metastatic process being involved in cell mobility; loss of cell-cell contact; interaction of cells with their environment, a process that is also relevant for tumor progression since cancer cells can modulate their microenvironment, as well as distant tissues, as seen in pre-metastatic niches; regulation of cell volume; apoptosis; angiogenesis [20, 56, 58–60]. Furthermore, it has already been established that integrins (adhesion proteins relevant to cell migration) and potassium channels are profoundly correlated [61]. Moreover, in several types of tumor cells, the expression of these potassium channels, as well as genes involved in their production, was observed to be increased, especially in the early stages, due to their importance in cell proliferation [57, 58, 62]. This may explain the rapid increase in potassium levels at the primary tumor at weeks 1 to 3 seen in Figs. 3 and 4.

Finally, phosphorus (P) and sulfur (S) presented similar distributions (as seen in Figs. 3 and 4), showing similarities even over the weeks. This is expected since both P and S are extremely necessary for cell division, a process continuously required for rapidly growing tumor tissues [63, 64].

## Conclusion

Although the importance of essential mineral elements in biological processes and tumor progression is well established in the literature, there is a scarcity of studies focusing on the investigation of distant tissues that could be conditioned by tumor cells to promote metastasis. In this context, the main objective of this study was to evaluate the elemental distribution of concentrations of several elements of known biological importance during tumor progression, focusing not only on the tumor tissue but also distant tissues.

This paper allowed the detection of significant changes in the elemental distribution of concentrations in distant tissues caused by the presence of a primary tumor, which could indicate a new aspect of pre-metastatic niche formation. It was also possible to observe the similarity between primary tumor and a tissue of non-origin, which is an unexpected and unprecedented result. Furthermore, it was possible to analyze and compare the elements’ concentrations distribution in a time-dependent manner for different tissues, such as primary tumor, liver, and lung, in Control and Tumor groups. The importance of elements for the biological processes of normal cells, as well as tumor cells during tumor progression, was also highlighted.

Perspectives of the present work include further investigations on the role of essential mineral elements in the formation of pre-metastatic niches. It is also our goal to explore the elemental signature presented by the primary tumor, as well as the greater similarity presented by the primary tumor with a tissue of non-origin.

Observations made throughout tumor progression allow for better comprehension of tumoral processes. Therefore, as achieved with the liver tissue, it would be interesting to collect data for the lung over the weeks of tumor progression to enable monitoring of tumor progression processes in this tissue over time. Another point to explore further is the use of our data to produce elemental maps (imaging technique) using the location data of elements (as described in section Data Preprocessing) to observe their spatial distribution in the tissues. Such results could provide further insights into tumoral processes and metallomics, allowing for the observation of possible proximity and associations with molecules and cellular structures, such as membrane proteins, or even proximity with structures, such as blood vessels.

The role of essential mineral elements as modulators of the tumor microenvironment and cell fate is a relevant aspect of tumor progression and this work is a contribution to this field. The description of elemental distribution patterns from an in vivo model of tumor progression described in this work highlights the complexity of tumoral metallomics and ionic equilibrium.

## Supplementary Information

**Additional file 1.** Supplementary figure.

## Data Availability

Additional Files, datasets and code used for data preparation and analysis are publicly available in the GitHub repository (https://github.com/SamellaSalles/elemental-distribution).

## Abbreviations

LLC: Lewis Lung Carcinoma
LNLS: Brazilian Synchrotron Light Source Laboratory
SOD: Superoxide Dismutase
ROS: Reactive Oxygen Species

## Footnotes

1 https://github.com/SamellaSalles/elemental-distribution
