# Supplementary material for: Elemental profiles in distant tissues during tumor progression
Source: BMC Cancer. 2023 Apr 6;23:322. doi: 10.1186/s12885-023-10782-w (PMC10080929; doi:10.1186/s12885-023-10782-w)
Supplement: Supplementary file 1 — Additional file 1. Supplementary figure. [file 12885_2023_10782_MOESM1_ESM.pdf]

Supplementary Information for

Elemental profiles in distant tissues during tumor progression

Samella Salles[1], Rebecca Salles[2], Mauro S. G. Pavão[3], Simone C. Cardoso[4] and Mariana P. Stelling[1*]

Mariana Paranhos Stelling
Email: mariana.stelling@ifrj.edu.br

This file includes:

Supplementary Figure S1

Supplementary Figure

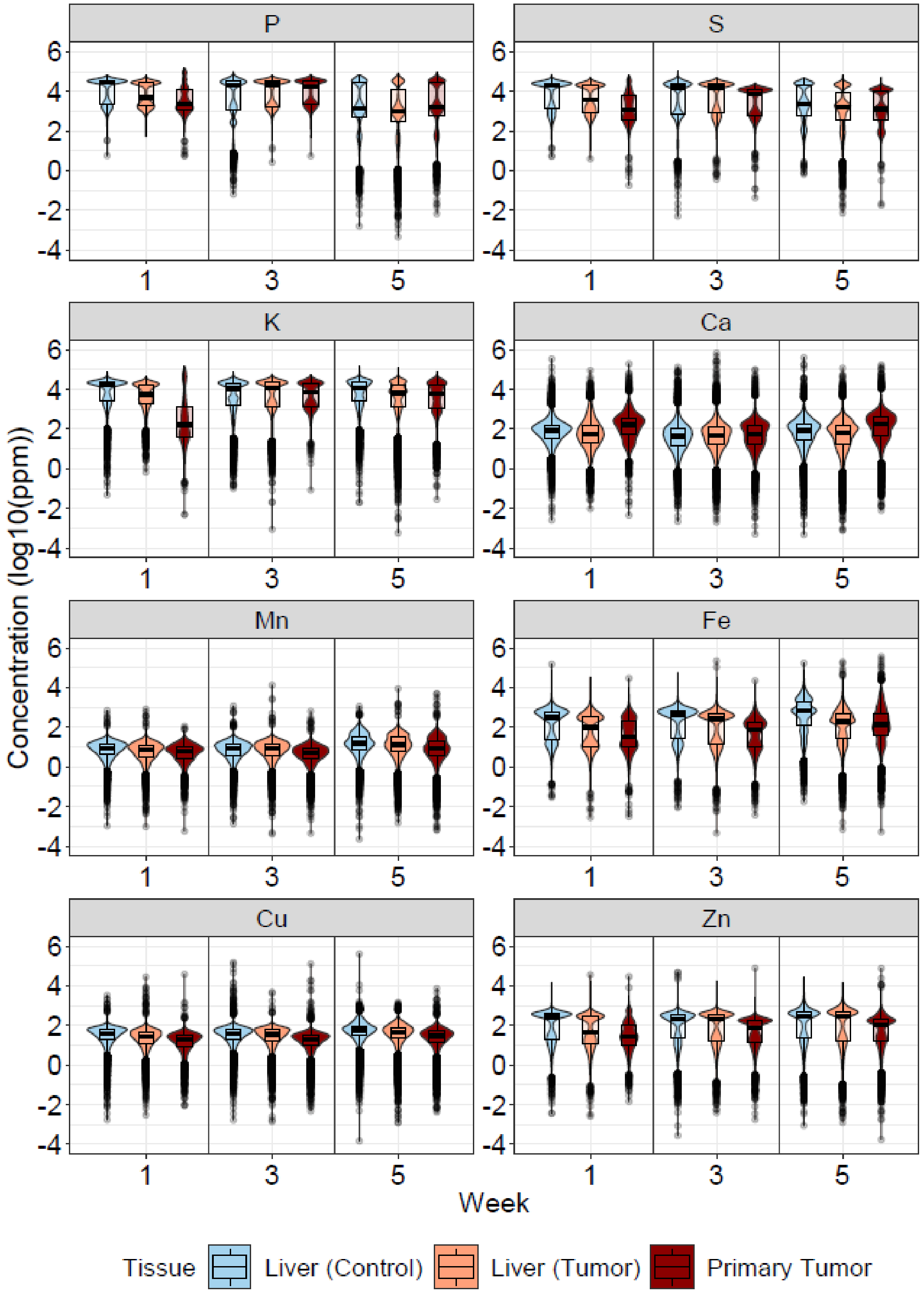

Figure S1. Comparison of the elements' distributions throughout the weeks (1, 3, and 5) in the liver (Control and Tumor groups) and primary tumor. Boxplots and violin plots in which light colors represent the liver, with light blue showing the Control group and light red the Tumor group. The primary tumor is represented in dark red.
